# Weighted Breaths: Exploring Biologic and Non-Biologic Therapies for Co-Existing Asthma and Obesity

**DOI:** 10.1007/s11882-024-01153-x

**Published:** 2024-06-15

**Authors:** Albert W. Pilkington, Bhanusowmya Buragamadagu, Richard A. Johnston

**Affiliations:** 1 Health Effects Laboratory Division, National Institute for Occupational Safety and Health, Centers for Disease Control and Prevention, United States Department of Health and Human Services, 1000 Frederick Lane, Morgantown, WV 26508-5402 USA; 2 Section of Pulmonary, Critical Care, and Sleep Medicine, Department of Medicine, School of Medicine, West Virginia University, Morgantown, WV USA; 3 Department of Physiology, Pharmacology, and Toxicology, School of Medicine, West Virginia University, Morgantown, WV USA

**Keywords:** Asthma, Biologic, Clinical trial, Forced expiratory volume in one second, Obesity, Probiotic

## Abstract

**Purpose of Review:**

To discuss the effectiveness of biologics, some of which comprise the newest class of asthma controller medications, and non-biologics in the treatment of asthma co-existing with obesity.

**Recent Findings:**

Our review of recent preliminary and published data from clinical trials revealed that obese asthmatics respond favorably to dupilumab, mepolizumab, omalizumab, and tezepelumab, which are biologics currently indicated as add-on maintenance therapy for severe asthma. Furthermore, clinical trials are ongoing to assess the efficacy of non-biologics in the treatment of obese asthma, including a glucagon-like peptide-1 receptor agonist, a Janus kinase inhibitor, and probiotics.

**Summary:**

Although many biologics presently indicated as add-on maintenance therapy for severe asthma exhibit efficacy in obese asthmatics, other phenotypes of asthma co-existing with obesity may be refractory to these medications. Thus, to improve quality of life and asthma control, it is imperative to identify therapeutic options for all existing phenotypes of obese asthma.

## Introduction

Asthma, a heterogenous, chronic lung disease, exists as two endotypes [T-helper cell type-2 (T_H_2) high and T_H_2 low], which can each be subdivided into multiple molecular phenotypes [1]. Despite the heterogeneity of asthma, endotypes of this disease share common symptoms, including cough, dyspnea, wheeze, persistent lung inflammation, variable expiratory airflow limitation, and airway hyperresponsiveness (AHR) [2]. Asthma afflicts children and adults, and globally, in 2019, there were 262 million asthmatics, which accounted for 21.6 million disability-adjusted life years (DALYs) [3]. Although the number of deaths attributed to asthma has decreased by 17.4% between 2010 and 2019, asthma-related morbidity continues to rise [3].

Obesity is the excessive or abnormal accumulation of adipose tissue in the body [4], and globally, in 2022, 160 million children and 890 million adults were obese [5]. Numerous sequelae are associated with obesity, including cardiovascular disease, non-alcoholic fatty liver disease, osteoarthritis, and type 2 diabetes [6]. In 2019, there were an estimated 5 and 160 million obesity-related deaths and DALYs, respectively, worldwide [7]. Body mass index (BMI), which is an indirect measure of body fat that is based on height and weight, is calculated by dividing weight in kilograms (kg) by the square of height in meters (m) and is used to establish the following weight categories for adults: underweight (< 18.5 kg/m^2^), normal weight (18.5–24.9 kg/m^2^), overweight (25–29.9 kg/m^2^), and obesity (≥ 30 kg/m^2^) [4]. However, in children, a weight category is assigned based on BMI relative to other children of the same age and sex [8].

Commencing in 1986 and continuing to the present day, epidemiologists have demonstrated that obesity increases the prevalence and incidence of asthma in children and adults [9–16]. In many, but not all studies, this relationship appears to be stronger in females as compared to males [17]. Since asthma is frequently over-diagnosed in both obese and non-obese individuals, it is improbable that the increased prevalence and incidence of asthma in obesity is the result of over-diagnoses in this population [18]. Consistent with epidemiological data, cluster analyses of adult asthmatics of varying nationalities have identified distinct clusters of either T_H_2 high or low asthmatics who are obese and predominately female [19–26]. In addition to increasing the prevalence and incidence of asthma in children and adults, obesity increases asthma severity and decreases quality of life and asthma control [27–32]. Underscoring the continued global increase in obesity and its impact on asthma morbidity, Liu et al. [33] reported that the number of asthma DALYs in overweight and obese individuals increased by 63.91% from 1990 to 2019. Although the overall number of global asthma deaths decreased between 2010 and 2019 [3], the number of asthma deaths specifically among overweight and obese individuals increased by 69.69% from 1990 to 2019 [33]. Given these data, it is unsurprising that total baseline health care costs are higher in obese as compared to normal-weight asthmatics [34].

As mentioned in the preceding paragraph, obese asthmatics have more severe asthma exacerbations and poorer asthma control. According to the 2020 Focused Updates to the Asthma Management Guidelines [35], the preferred treatment for persistent asthma in individuals twelve years of age and older is combination therapy: an inhaled corticosteroid with either a short- or long-acting β_2_-adrenergic receptor agonist. However, if these medications are insufficient to achieve satisfactory asthma control, the Guidelines recommend that other medications be added to the treatment regimen, including long-acting muscarinic antagonists, oral corticosteroids, or biologics [35]. Treatment of obese asthmatics with pharmacological interventions is challenging since they are often refractory to standard asthma medications. For example, overweight and/or obese asthmatics, whether children or adults, exhibit poor responsiveness to corticosteroids as compared to normal-weight asthmatics [21, 36, 37]. Obese asthmatics also do not respond as favorably as normal-weight asthmatics to combination therapy: inhaled corticosteroids and long-acting β_2_-adrenergic receptor agonists [37, 38].

Obese asthmatics who achieve weight loss through either diet and/or surgery demonstrate improved lung function, quality of life, and asthma control as well as a decrease in airway responsiveness [39–43]. Because weight loss is difficult to maintain and because obese asthmatics respond poorly to standard asthma medications [21, 36–38, 44], it is essential to identify new pharmacological interventions to improve the quality of life for obese asthmatics. This is particularly important since weight loss via bariatric surgery reduces airway responsiveness in obese subjects with late-onset non-atopic asthma while it has no effect on airway responsiveness in obese subjects with early-onset atopic asthma [45].

Biologics are products derived from living organisms that can be used for multiple purposes, including the diagnosis, prevention, or treatment of disease [46, 47], and in 2003, the United States (U.S.) Food and Drug Administration (FDA) approved the first biologic, omalizumab, for the treatment of asthma [48]. Given that (1) obese asthmatics are often refractory to standard asthma medications [21, 36–38] and (2) there has been a recent explosion of biologics potentially available for the treatment of asthma, we shall, in the remainder of this review, discuss the effectiveness of biologics in the management of asthma co-existing with obesity. We shall, for obese asthma, review biologics that fall into the following categories: anti-immunoglobulin (Ig) E, anti-T_H_2, anti-alarmin, and those that do not specifically fall into any of the prior categories. Finally, we shall also discuss potentially novel medications, other than biologics, that may be useful for the treatment of obese asthma.

## Biologics and Obese Asthma

### Anti-IgE

#### Omalizumab

Omalizumab, a humanized anti-IgE monoclonal antibody, is currently recommended as add-on therapy for patients six years of age and older with severe allergic asthma [49]. By binding to circulating IgE, omalizumab prevents IgE from engaging its high-affinity receptor, FcεRI, on the surface of basophils and mast cells, which degranulate when antigen cross-links neighboring IgE-FcεRI complexes [50, 51]. When basophils and mast cells are prevented from degranulating, many of the deleterious mediators, including cytokines, histamine, leukotrienes, and proteases, which promote allergic inflammation, fail to enter the extracellular milieu [51, 52].

In 2019, Oliveira et al. [53] reported that obese individuals with severe asthma administered omalizumab every two to four weeks over a twelve-month period exhibited significant improvement in lung function [i.e., forced expiratory volume in one second (FEV_1_)] and asthma control. The administration of omalizumab also decreased the number of asthma exacerbations and the prescribed dose of inhaled corticosteroids. As compared to placebo, Geng et al. [54] demonstrated that omalizumab essentially had the same effects in obese individuals with moderate-to-severe allergic asthma as those of Oliveira et al*.* [53]. In contrast, Sposato et al. [55] reported that obesity reduced the effectiveness of omalizumab in severe allergic asthmatics while Gibson et al. [56] revealed that it was significantly more probable that obese as compared to non-obese allergic asthmatics would be classified as non-responders to omalizumab. Although typically reserved for severe allergic asthma, administration of omalizumab to non-atopic asthmatics, the majority of whom were obese, reduced emergency room visits, hospitalizations, and corticosteroid use [57]. Thus, omalizumab could become a viable controller medication for select non-atopic obese asthmatics, yet more rigorous studies are needed.

### Anti-T_H_2

#### Dupilumab

To independently initiate signal transduction and consequently sequelae of atopic inflammation via the Janus kinase-signal transducer and activator of transcription (JAK-STAT) pathway, interleukin (IL)-4 and IL-13, which are T_H_2 cytokines, utilize, in part, the IL-4 receptor subunit alpha (IL-4Rα) [58]. Specifically, IL-4 signals via the type I IL-4 receptor (IL-4R) complex, which is a heterodimer consisting of IL-4Rα and the cytokine receptor common subunit gamma (γc) while IL-13 signals via the type II IL-4R complex, which is also a heterodimer but consists of IL-4Rα and the IL-13 receptor subunit alpha-1 (IL-13Rα1) [58]. IL-4 is required for the differentiation of T_H_2 cells, suppression of T regulatory (T_reg_) cells, IgE production in B cells, and adhesion of eosinophils to the walls of blood vessels while IL-13 induces airway smooth muscle contraction and proliferation and increases expression of FcεRI on the surface of mast cells, eotaxin and mucin in bronchial epithelial cells, and IgE in B cells [59–61]. Following antigen sensitization and challenge, Dahm et al. [62] demonstrated that bronchoalveolar lavage (BAL) IL-4 and IL-13 were significantly greater in mice obese because of a genetic deficiency in carboxypeptidase E (*Cpe*^*fat*^ mice) as compared to lean wild-type mice. However, in human subjects, neither sputum IL-4 nor IL-13 messenger ribonucleic acid (mRNA) expression were different between lean and obese asthmatics [63].

Dupilumab, a human IgG_4_ monoclonal antibody, antagonizes IL-4 and IL-13 signal transduction by binding to IL-4Rα, which is expressed by hematopoietic and non-hematopoietic cells [64, 65]. According to the Global Initiative for Asthma [49], dupilumab is recommended as add-on therapy for (1) individuals who are six years of age and older with severe eosinophilic/T_H_2-high asthma or (2) adolescents and adults that require maintenance treatment with oral corticosteroids. Presently, preliminary data exists from one randomized, double blind, placebo-controlled study in which investigators examined the impact of BMI on the effectiveness of dupilumab in a cohort of patients with uncontrolled, moderate-to-severe asthma [66, 67]. Specifically, regardless of BMI, dupilumab, as compared to placebo, significantly improved FEV_1_ and decreased the annualized rate of severe asthma exacerbations.

#### Mepolizumab and Benralizumab

**Fig. 1:**
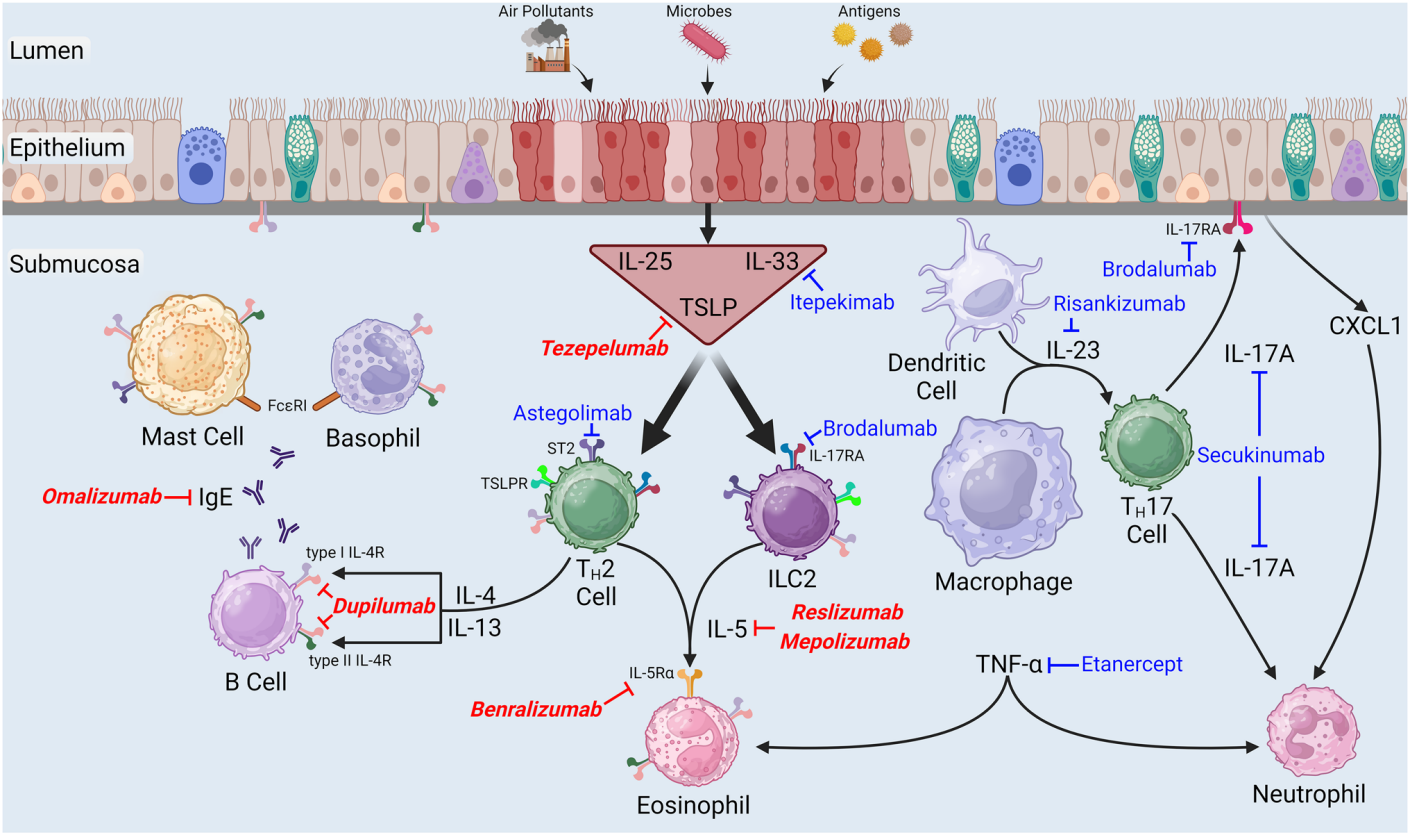
Exposure of the luminal surface of the respiratory epithelium to injurious stimuli, including air pollutants, microbes, or enzymatically-active antigens leads to the release of alarmins [interleukin (IL)-25, IL-33, and thymic stromal lymphopoietin (TSLP)] from epithelial cells and the initiation of multiple inflammatory cascades, which are important in the pathogenesis of asthma. By engaging their respective receptors described in the body of this review, these alarmins stimulate the release of T-helper cell type-2 (T_H_2) cytokines (IL-4, IL-5, and IL-13) from group 2 innate lymphoid cells (ILC2) and T_H_2 cells. Once released into the extracellular space, these T_H_2 cytokines subsequently bind their corresponding receptor subunits, which are part of a heterodimeric receptor complex located on the surface of various hematopoietic and non-hematopoietic cells. Immunoglobulin (Ig) E, which is released from B cells in response to IL-4 and IL-13, binds its high-affinity receptor, FcεRI, on the surface of basophils and mast cells. Following antigen cross-linking of IgE-FcεRI complexes on the surface of basophils and mast cells, deleterious mediators of allergic inflammation are secreted into the extracellular milieu. Activated dendritic cells and macrophages secrete IL-23, which stimulates the release of IL-17A from T_H_17 cells. IL-17A, in turn, initiates the release of chemokine (C-X-C motif) ligand 1 (CXCL1), a chemotactic cytokine for neutrophils, from epithelial cells [99, 152], which leads to neutrophil migration to the air spaces. Finally, tumor necrosis factor (TNF)-α, which is increased in asthmatic airways, causes eosinophil and neutrophil chemotaxis [104]. The name of each biologic discussed in this review has been placed next to its molecular target, and those biologics in bold italicized red font are currently approved by the United States Food and Drug Administration as add-on maintenance therapy for severe asthma. Please note that this figure does not comprehensively illustrate (1) cytokine release from or (2) the presence of cytokine receptors on each cell type shown in this figure. This figure was created using BioRender (Toronto, Ontario, Canada)

The biological response to inhaled asthma stimuli, including air pollutants, antigens, and viruses, is partially characterized by secretion of IL-5, a T_H_2 cytokine, from T_H_2 lymphocytes and/or group 2 innate lymphoid cells (ILC2) (Fig. 1) [68–70]. Once released into the extracellular space, IL-5 can bind the IL-5 receptor subunit alpha (IL-5Rα) on the surface of eosinophils, an event that facilitates interaction with the cytokine receptor common subunit beta (βc), which subsequently leads to eosinophilopoiesis and eosinophil maturation and survival [70, 71]. In asthma, eosinophils drive AHR, mucus production, tissue injury, and airway remodeling [72]. Since sputum IL-5 and submucosal eosinophils are greater in obese as compared to lean asthmatic human subjects [63, 73], it is reasonable to speculate that currently available monoclonal antibodies directed against either IL-5 (mepolizumab and reslizumab) or IL-5Rα (benralizumab) for severe eosinophilic asthma could be beneficial add-on therapy for obese asthmatics [49, 74].

In a *post-hoc* meta-analysis, Albers et al. [75] reported that regardless of BMI mepolizumab as compared to placebo (1) decreased blood eosinophil counts and the annual rate of asthma exacerbations and (2) increased asthma control and pre-bronchodilator FEV_1_ in patients twelve years of age and older with severe eosinophilic asthma. Consistent with Albers et al*.* [75], preliminary data from Da Cunha et al. [76] demonstrated that mepolizumab administration over a twelve-month period effectively decreased blood eosinophil counts and the number of asthma exacerbations in eleven obese asthmatics.

To date, only preliminary data from clinical trials evaluating the effectiveness of benralizumab in obese asthmatics has been made publicly available, and the authors of these reports demonstrate that benralizumab is less effective in obese as compared to non-obese asthmatics. In the first study, a *post-hoc* pooled analysis of data extracted from the SIROCCO and CALIMA clinical trials was performed, and as compared to placebo, subcutaneous administration of benralizumab to obese adults with severe, uncontrolled eosinophilic asthma numerically caused (1) an improvement in pre-bronchodilator FEV_1_ and (2) a reduction in the number of asthma exacerbations [77–79]. However, in this same study, benralizumab significantly improved FEV_1_ and decreased the number of asthma exacerbations in normal/underweight and overweight asthmatics [78]. In the second study, Nanzer et al. [80] reported that obesity impaired the beneficial effects of benralizumab in patients with severe eosinophilic asthma. Taken together, it is unclear if benralizumab significantly improves lung function and asthma control in obese asthmatics. Nevertheless, these data certainly provide a strong rationale to pursue further clinical trials evaluating the efficacy of benralizumab in obese individuals with severe eosinophilic asthma.

### Anti-Alarmin

Following activation of pattern recognition receptors on the surface of airway epithelial cells or in response to cell injury or death initiated by diverse stimuli, including air pollution, microbes, or enzymatically-active antigens, epithelial cells release constitutively expressed peptides and proteins (i.e., alarmins), which serve as intercellular defense signals to heighten host defenses (Fig. 1) [81–83]. Regarding asthma, the most widely studied alarmins include IL-25, IL-33, and thymic stromal lymphopoietin (TSLP), which can all drive allergic inflammation via stimulation of T_H_2 cells and ILC2 [82, 83]. Sputum IL-25 is greater in obese as compared to lean asthmatics while BAL IL-33 and TSLP are greater in obese as compared to lean mice with experimental asthma [63, 69, 84]. Neutralizing antibodies against TSLP or IL-1 receptor-like 1 (IL1RL1), which is the receptor for IL-33 and which is also known as suppression of tumorigenicity 2 (ST2), reduced phenotypic features of T_H_2 inflammation induced by ILC2 in obese mice with antigen-induced lung inflammation without impacting features of neutrophilic inflammation [84]. However, Mathews et al. [69] demonstrated that an anti-ST2 antibody reduced (1) BAL neutrophils, (2) features of inflammation induced by ILC2, and (3) increases in airway responsiveness in obese mice with experimental asthma induced by the non-atopic asthma stimulus, ozone (O_3_). Taken together, these data suggest that anti-alarmin biologics may be effective in the treatment of obese asthma.

#### Brodalumab

Brodalumab is a human IgG_2_ monoclonal antibody with a high affinity for IL-17 receptor A (IL-17RA), which is used, in part, by IL-25 in addition to IL-17A, IL-17C, IL-17F, and the IL-17A/F heterodimer to transduce intracellular signals [85, 86] Although currently approved to treat moderate-to-severe plaque psoriasis that is refractory to other therapies [85], Busse et al. [86] executed a phase 2a, randomized, double-blind, placebo-controlled, clinical trial to assess the effectiveness of brodalumab as a treatment for moderate-to-severe asthma. However, as compared to placebo, brodalumab did not improve lung function or symptoms scores in the full study population. In a separate interventional clinical trial, brodalumab demonstrated no efficacy on asthma control in adult asthmatics specifically exhibiting high bronchodilator reversibility [87]. Of importance, subjects in neither study were recruited according to BMI status. Thus, given that IL-17A and IL-25, which both use, in part, IL-17RA to exert their biological effects, are significantly greater in sputum of obese as compared to non-obese asthmatics [63], future studies focusing on the effectiveness of brodalumab in obese asthma is warranted.

#### Itepekimab and Astegolimab

Itepekimab is an IgG_4P_ monoclonal antibody against IL-33, and in 2021, Wechsler et al. [88] reported the results of a phase 2 clinical trial evaluating the effectiveness of itepekimab in the treatment of moderate-to-severe asthma. As compared to placebo, itepekimab monotherapy significantly improved asthma control and pre-bronchodilator FEV_1_. Nevertheless, the investigators did not specifically examine the impact of BMI on the effectiveness of itepekimab. The efficacy of astegolimab, a human IgG_2_ monoclonal antibody directed against ST2, was assessed for the treatment of severe asthma in a phase 2b, randomized, placebo-controlled, double-blind clinical trial in which thirty-six percent of the patients had a BMI greater than 30 kg/m^2^ [89]. Over the fifty-two-week trial, astegolimab, when compared to placebo, improved quality of life and reduced the number of asthma exacerbations only in participants with low numbers of blood eosinophils. Although thirty-six percent of subjects in this trial were obese, the investigators did not specifically examine the effectiveness of astegolimab in their obese patients. Thus, it is of interest to determine the specificity of astegolimab in the treatment of obese asthma with neutrophilic inflammation given the effectiveness of an anti-ST2 antibody in a mouse model of obese asthma that is dominated by neutrophils [69].

#### Tezepelumab

To initiate intracellular signaling, TSLP requires a heterodimeric receptor complex consisting of cytokine receptor-like factor 2 (CRLF2 or TSLPR) and the IL-7 receptor subunit alpha (IL-7Rα) [90]. Tezepelumab, a human monoclonal anti-TSLP antibody, is indicated as add-on treatment for individuals twelve years of age and older with severe asthma [49, 91]. To exert its beneficial effects, tezepelumab binds free TSLP, which is then prevented from subsequently binding TSLPR [91]. Preliminary data extracted from the DESTINATION, NAVIGATOR, and PATHWAY clinical trials illustrate that, regardless of an asthmatic’s baseline BMI, tezepelumab administration reduced the annualized asthma exacerbation rate [92–96]. Thus, this is promising evidence that the newest asthma controller medication, tezepelumab, may be effective in obese asthmatics.

### Miscellaneous Biologics

#### Secukinumab

IL-17A, an extensively studied pro-inflammatory cytokine, is produced by a plethora of cells, including T_H_17, γδ T, invariant natural killer T, lymphoid-tissue inducer-like, and Paneth cells as well as ILC3 [97, 98]. Engagement of an IL-17A homodimer or an IL-17A/F heterodimer with the IL-17 receptor complex, which consists of IL-17RA and IL-17RC, leads to increased expression of neutrophil chemotactic cytokines, granulopoiesis factors, acute phase proteins, and pro-inflammatory cytokines such as IL-1β and TNF-α [99]. Sputum IL-17A is greater in obese as compared to non-obese asthmatics while neutralization of IL-17A in genetically obese mice reduced O_3_-induced increases in airway responsiveness in addition to BAL keratinocyte chemoattractant (KC) and neutrophils [63, 100].

A single clinical trial evaluating the effectiveness of secukinumab, an anti-IL-17A human IgG_1_κ monoclonal antibody, in poorly controlled asthma was terminated prior to completion with the caveat that future clinical trials involving this biologic require an extensive overhaul, including modifications to the study design, endpoints, and population as well as the use of a different anti-IL-17A antibody [101, 102]. Thus, if further clinical trials with secukinumab are executed in asthmatics, it would be crucial to stratify subjects via BMI given the previously aforementioned human and animal subject data concerning the potential importance of IL-17A in obese asthma [63, 100].

#### Etanercept

The deleterious effects of TNF-α in inflammatory diseases, including asthma and obesity, are well established [103–105]. Obesity increases serum TNF-α in both humans and mice [106, 107], and a polymorphism in the promoter region of the human gene (TNF), which leads to increased TNF expression, is coupled to a stronger association of obesity with asthma, particularly non-atopic asthma [108, 109]. However, in obese mice genetically deficient in TNF-α, the severity of increases in airway responsiveness induced by O_3_ were enhanced, which implies a protective effect of TNF-α in this animal model of non-atopic asthma [110]. In contrast, Kim et al. [111] reported that neutralization of TNF-α with a polyclonal antibody decreased airway responsiveness in antigen sensitized and challenged mice. Consistent with the results from pre-clinical animal studies, the effectiveness of etanercept, a humanized soluble TNF receptor fusion protein that neutralizes the effects of TNF-α, has been inconsistent in the treatment of asthma [112]. For example, in an open label uncontrolled clinical study involving fifteen patients, Howarth et al. [113], despite demonstrating that etanercept significantly improved lung function and decreased asthma symptoms and airway responsiveness, reported that etanercept paradoxically led to asthma exacerbations and respiratory tract infections in 52.9 and 58.8% of participants, respectively. In a randomized, double-blind, placebo-controlled clinical trial, etanercept failed to improve pre-bronchodilator FEV_1_, quality of life, or asthma control in adults with moderate-to-persistent asthma [114]. Finally, Berry et al. [115] reported, as compared to placebo, that subcutaneous administration of etanercept twice weekly over a ten-week period, reduced responsiveness to methacholine, increased pre-bronchodilator FEV_1_, and improved quality of life. It is important to note that none of these studies stratified patients by BMI. If, in the future, studies are designed to specifically evaluate the efficacy of etanercept in obese asthma, caution must be taken since anti-TNF-α therapy is associated with statistically significant weight gain [116].

#### Risankizumab

IL-23, a member of the IL-12 family of cytokines, consists of two subunits, IL-12p40, which it shares with IL-12, and IL-23p19, and is secreted by activated dendritic cells and macrophages (Fig. 1) [117, 118]. The biological effects exerted by IL-23, including differentiation of naïve T cells to T_H_17 cells, proliferation and survival of T_H_17 cells, and stimulation of IL-17A release from T_H_17 cells, manifest following engagement of IL-23 with its receptor, which also consists of two subunits [IL-12 receptor subunit beta-1 (IL-12Rβ1) and IL-23 receptor (IL-23R)] [119–122]. Obesity and asthma, independently, increase serum IL-23 in human subjects [123, 124], BAL IL-23 is increased to a greater extent in obese as compared to lean mice following exposure to O_3_ [100], and inhibition of IL-17A, whose expression can be induced by IL-23, reduces increases in airway responsiveness and BAL KC and neutrophils induced by acute exposure to O_3_ [100, 125]. Contrary to this evidence supporting a role for IL-23 in the pathogenesis of obese asthma, Brightling et al. [126] reported that, as compared to placebo, administration of risankizumab, a humanized IgG_1_ monoclonal antibody, which binds to the p19 subunit of IL-23, decreased the time to the first asthma worsening after treatment commenced, increased the annualized rate of asthma worsening, and had no effect on FEV_1_ or sputum eosinophils or neutrophils [127]. A subgroup analysis of the participants stratified by BMI also revealed that risankizumab was ineffective, as compared to placebo, at lengthening the time to the first asthma worsening [126].

## Non-Biologics and Obese Asthma

### Metformin

Metformin, a biguanide, is a first-line medication for the treatment of hyperglycemia in individuals with type 2 diabetes [128], and in adults with both asthma and type 2 diabetes, use of metformin is associated with a decreased number of asthma-related emergency room visits and hospitalizations [129]. However, Shore et al. [130] demonstrated that metformin administration to mice obese because of a genetic deficiency in the long isoform of the leptin receptor (Ob-Rb; *db*/*db* mice) had no effect on lung inflammation or increases in airway responsiveness induced by O_3_. In contrast, Guo et al. [131] reported that metformin administration decreased BAL IL-4 and TNF-α and lung inflammatory cell infiltrates but increased the frequency of immunosuppressive T_reg_ cells in antigen-sensitized and challenged CD-1 mice with dietary obesity. The ratio of T_reg_ to T_H_17 cells is reduced in obese subjects with type 2 diabetes, a phenomenon driven by a reduction in the frequency of T_reg_ cells [132]. Thus, restoring this imbalance, potentially through metformin, may offer a new strategy to blunt the pro-inflammatory effects of T_H_17 cells, and consequently, alleviate symptoms in atopic obese asthmatics.

### Semaglutide

Glucagon-like peptide-1 receptor (GLP-1R) agonists, including semaglutide, were initially approved by the U.S. FDA for the treatment of type 2 diabetes yet are now available for chronic weight management [133]. Recent data illustrate the potential for GLP-1R agonists to treat obese asthma. First, Toki et al. [84] demonstrated that treatment of genetically obese mice with liraglutide, a GLP-1R agonist, reduced increases in airway responsiveness, BAL T_H_2 cytokines (IL-5 and IL-13), eotaxin, and eosinophils in addition to BAL neutrophils and neutrophil chemotactic cytokines [IL-17, KC, and lipopolysaccharide-induced CXC chemokine (LIX)] following sensitization and challenge with *Alternaria alternata* extract. Second, patients with both asthma and type 2 diabetes and with a mean BMI of 39.5 ± 8.6 kg/m^2^ that were prescribed GLP-1R agonists exhibited fewer asthma exacerbations as compared to patients prescribed other classes of diabetic medications [134]. To that end, semaglutide is presently undergoing evaluation in a randomized, double-blind, placebo-controlled clinical trial to assess its effectiveness on asthma control in obese adults with persistent asthma [135].

### Povorcitinib

Over fifty cytokines, including IL-4, IL-5, IL-13, IL-23, and TSLP, which we previously discussed in this review, transduce intracellular signaling via proteins belonging to the JAK-STAT family [71]. Consistent with the role of the aforementioned cytokines driving the migration of eosinophils to the lungs in animal models of asthma [84, 136–138], inhibiting JAK family members that are activated upon engagement of these cytokines with their receptors decreases BAL eosinophils in antigen sensitized and challenged mice [139–142]. Currently, a phase 2 interventional clinical trial is ongoing to assess the effect of povorcitinib, an oral small-molecule inhibitor of JAK1, on pre-bronchodilator FEV_1_ in individuals with inadequately controlled moderate-to-severe asthma [143]. From publicly available data, however, it is unclear if the participants in this trial will be stratified by BMI. Nevertheless, Lyu et al. [144] recently demonstrated that reticuline, an inhibitor of JAK2-STAT3 and NF-κB signaling, significantly decreased, in mice, antigen-induced increases in airway responsiveness, BAL IL-5 and IL-17A, and the number of BAL and lung tissue eosinophils and neutrophils [145]. Therefore, selective inhibitors of JAK-STAT family members could be beneficial in the treatment of obese asthma. Notwithstanding, use of JAK-STAT inhibitors in asthma co-existing with obesity should be approached with caution since activation of specific JAK-STAT family members can attenuate the severity of obesity-induced sequelae, including atherosclerosis and hepatic steatosis [146, 147].

### Probiotics

According to Berg et al. [148], the microbiome is a community of microorganisms and their accompanying internal and external structural elements that exude unique physiochemical properties while occupying a distinct environment. Interestingly, the BAL, fecal, nasal, and oral microbiomes of obese asthmatics are uniquely different from those of non-obese asthmatics and obese non-asthmatics [149], which could influence the course of the obese asthma phenotype, since manipulation of the gut microbiome in obese *db*/*db* mice with antibiotics decreases the severity of O_3_-induced increases in airway responsiveness [150]. Thus, altering the gut microbiome in obese asthmatics with supplements that maintain a healthy community of microorganisms (i.e., probiotics) may be a beneficial therapeutic intervention for these individuals. Indeed, an interventional clinical trial, which is scheduled to be completed in March of 2025, will provide data, in part, to determine if oral probiotics improve lung function, quality of life, and asthma control in obese asthmatics [151].

## Conclusions

The expanding arsenal of biologics presents promising options for obese asthmatics who are often poorly responsive to standard asthma medications. Preliminary or published data illustrate that select, currently available biologics indicated as add-on maintenance therapy for severe asthma (dupilumab, mepolizumab, omalizumab, and tezepelumab) improve lung function and asthma control and/or reduce asthma exacerbations in obese asthmatics. In addition, the effectiveness of non-biologics, including povorcitinib, probiotics, and semaglutide, in obese asthma are presently being assessed. Because each of these pharmacological interventions have different mechanisms of action, this offers a diverse approach to the management of obese asthma. However, since obese asthma encompasses diverse molecular phenotypes, it is imperative that new therapeutics continuously be identified to successfully treat those obese asthma phenotypes, which may be refractory to medications that already effectively treat other phenotypes of this disease.
